# Successful Management of Acute Obscure Gastrointestinal Bleeding (OGIB) Using Long Tube-Directed Hemostasis: A Case Report

**DOI:** 10.7759/cureus.84251

**Published:** 2025-05-16

**Authors:** JuDong Zhang, YiFang Hsieh, Jing Xu

**Affiliations:** 1 Department of Surgery, Tianjin Union Medical Center, The First Affiliated Hospital of Nankai University, Tianjin, CHN; 2 School of Medicine, Nankai University, Tianjin, CHN

**Keywords:** acute gastrointestinal bleeding, case report, intervention, long tube, obscure gastrointestinal bleeding

## Abstract

Obscure gastrointestinal bleeding (OGIB) is characterized by persistent or recurrent bleeding without a clear source despite standard endoscopic and radiographic investigation. Acute, severe OGIB presents a significant therapeutic challenge, particularly when hemodynamic instability precludes invasive diagnostic procedures. A 74-year-old Asian man presented with acute, severe OGIB and massive hematochezia. Initial laboratory tests revealed severe anemia (hemoglobin 31 g/L) and hypofibrinogenemia (0.95 g/L). Despite negative findings on gastroscopy, colonoscopy, abdominal CT, and angiography, and despite aggressive resuscitation with fluids, blood products, and hemostatic agents, bleeding persisted. A long tube was placed under fluoroscopic guidance, and bloody aspirate confirmed its distal jejunal position. Localized administration of thrombin and Yunnan Baiyao via the long tube achieved hemostasis within 24 hours, and the patient's hemoglobin stabilized. He was discharged after 10 days. This case demonstrates the successful use of long tube-directed hemostatic therapy in acute, severe OGIB when conventional methods are inconclusive and further diagnostic evaluation is limited by the patient's clinical condition. This approach may represent a valuable alternative for achieving hemostasis and bridging patients to definitive therapy. Further research is warranted to confirm the efficacy and safety of this technique.

## Introduction

Obscure gastrointestinal (GI) bleeding (OGIB) poses a significant diagnostic and therapeutic challenge, characterized by persistent or recurrent GI bleeding without a clear source despite standard endoscopic and radiographic investigation, including small bowel assessment [1]. This often necessitates multiple diagnostic procedures, increasing patient burden and healthcare costs. OGIB accounts for approximately 5% of GI bleeding cases, with small bowel vascular ectasias frequently implicated [2]. While advances in capsule endoscopy, enteroscopy [3], and angiography [4] have improved diagnostic yields, pinpointing the bleeding site can remain elusive, hindering targeted interventions [5]. This diagnostic uncertainty, compounded by the potential for hemodynamic instability in acute settings, necessitates innovative approaches to management. Traditional management of acute OGIB focuses on hemodynamic stabilization with fluid resuscitation, blood transfusions [1], and occasionally, empirical hemostatic agents [2]. However, definitive therapy requires identification and treatment of the underlying source.

Here, we report a case in which long tube-directed hemostatic therapy-including localized administration of thrombin and Yunnan Baiyao-achieved successful bleeding control in a patient with acute OGIB, when conventional diagnostics were inconclusive and further intervention was limited by clinical instability.

## Case presentation

**Figure 1:**
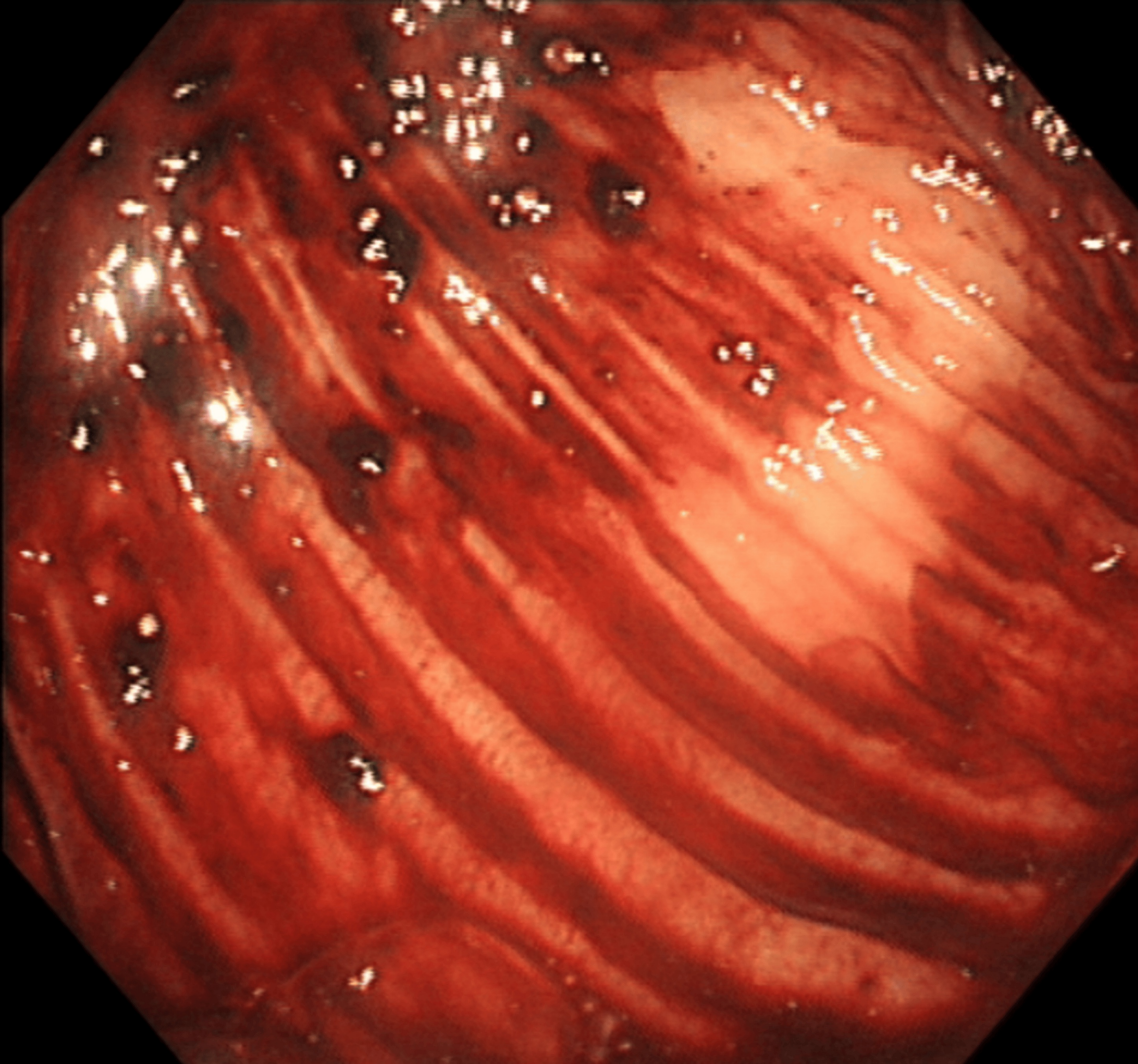
Colonoscopy result

**Figure 2:**
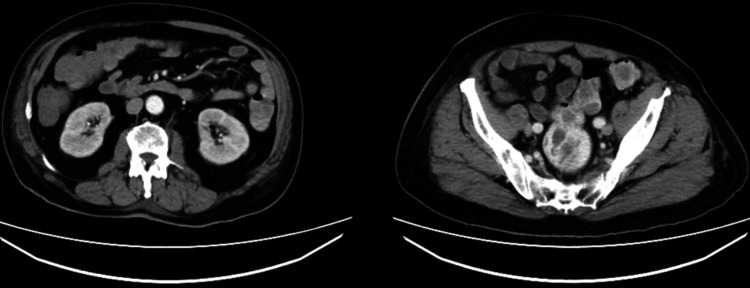
Abdominal CT reveals no active bleeding

A 74-year-old man presented to our institution with a one-day history of hematochezia, describing five days of bloody bowel movements, totaling approximately 800 mL of blood loss with clots. Accompanying symptoms included dizziness, weakness, and palpitations, but no nausea or vomiting. The patient denied recent use of non-steroidal anti-inflammatory drugs (NSAIDs). There were no signs of systemic vasculitis on physical examination or laboratory evaluation, including absence of rash, arthralgia, or abnormal autoimmune markers. The symptoms started five days prior to admission and worsened within the 24 hours before presentation. He appeared lethargic and pale, with conjunctival pallor, tachycardia (HR: 125 bpm), hypotension (BP: 92/65 mmHg), and tachypnea (RR: 22 breaths/min). His abdomen was soft and non-tender, with normal bowel sounds (8/min). Initial labs revealed severe anemia (hemoglobin 31 g/L) and hypofibrinogenemia (0.95 g/L). Gastroscopy and colonoscopy, while negative for an obvious bleeding source, revealed dark red blood in the cecum (Figure 1), suggesting a possible small bowel origin. Abdominal CT (Figure 2) and angiography were also negative for active bleeding. Upon admission, a central venous catheter (CVC) was placed via the internal jugular vein. The patient was made nil per os (NPO) and received aggressive fluid resuscitation with succinylated gelatin and hydroxyethyl starch, blood transfusions (plasma and packed red blood cells), and hemostatic therapy with thrombin and ethamsylate. Given the urgency and limited options at the time, gelatin and hydroxyethyl starch were used cautiously under close monitoring, despite potential concerns in patients with renal risk. Albumin and fibrinogen were also administered to correct deficiencies. Additionally, a continuous infusion of octreotide and omeprazole was initiated.

**Figure 3:**
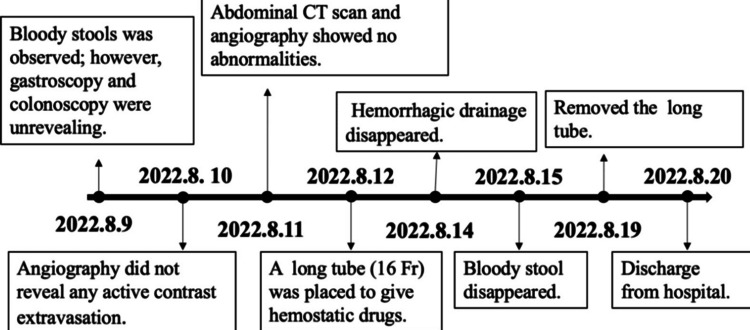
Treatment timeline

**Figure 4:**
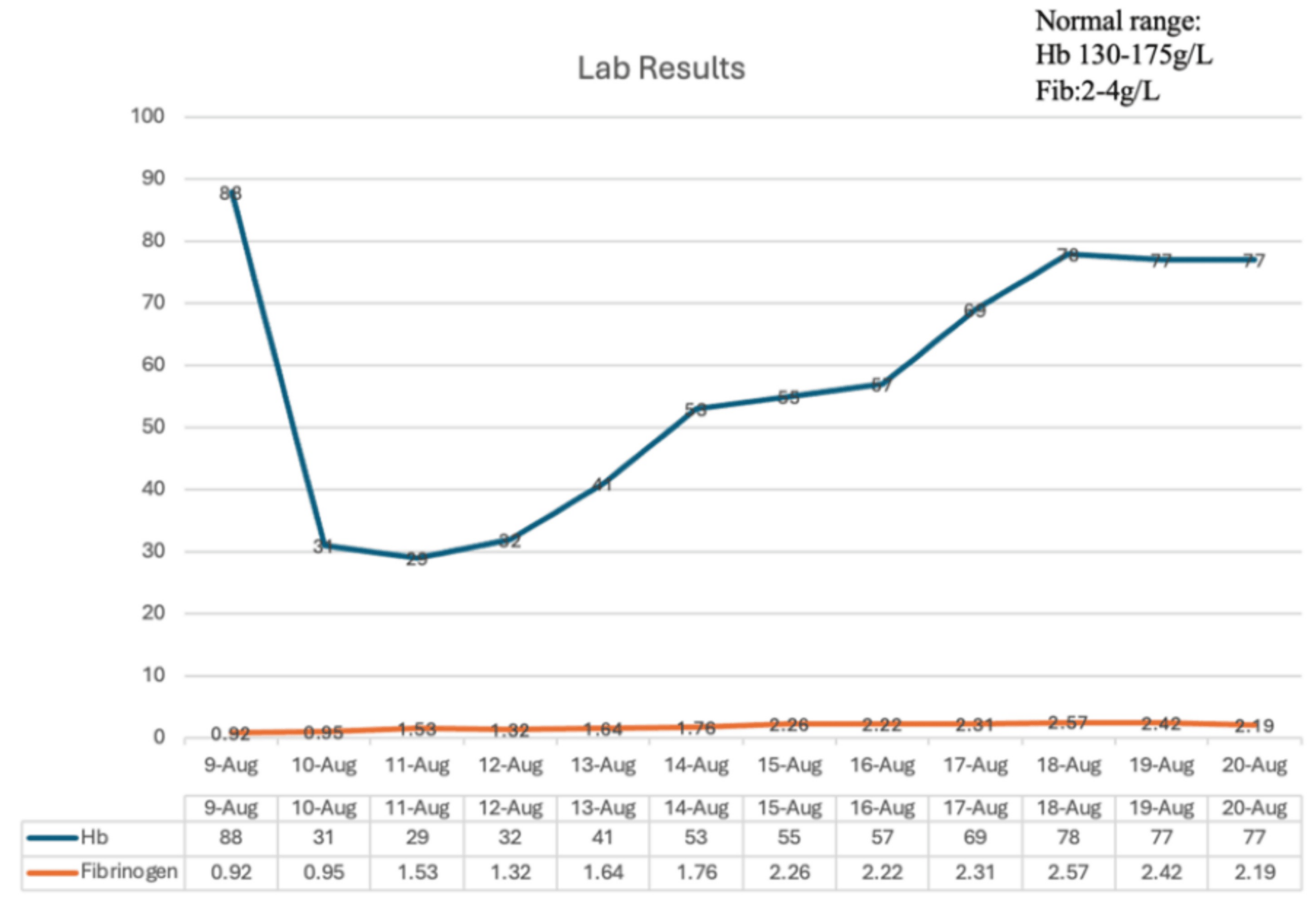
Hhemoglobin (Hb) levels and fibrinogen (fib) alteration

Despite these measures, the patient continued to have hematochezia the following day, with persistent severe anemia (hemoglobin 29 g/L). Repeat angiography showed a normal superior mesenteric artery and attenuated distal vessels without extravasation. Though small bowel bleeding was highly suspected, due to his hemodynamic instability, he was unable to tolerate enteroscopy, thus prompting placement of a long intestinal tube for localized hemostatic therapy under fluoroscopic guidance. A standard long intestinal decompression tube was used and placed by the interventional team. The long tube was advanced into the distal jejunum, confirmed by the aspiration of bloody fluid, allowing for targeted delivery of hemostatic agents. Thrombin (20,000 U) and Yunnan Baiyao (a proprietary Chinese hemostatic powder, 4 pills, 1 g) were administered via the tube every 12 hours (q12h) for seven days. Within 24 hours, his heart rate normalized, and hemoglobin levels began to rise. Two days later, the drainage cleared, and hematochezia resolved. After a week of continued treatment, his hemoglobin stabilized at 88 g/L, and he was discharged. The patient's total hospital stay was 10 days. During this time, he received 45 units of thrombin, 23 units of albumin, 16 units of prothrombin complex concentrate, 76 units of fibrinogen, 38 units of blood coagulation factor, 12 units of ethamsylate, 4,550 mL of plasma, and 5,200 mL of packed red blood cells. The patient's total hospital stay was 10 days. The detailed diagnostic and therapeutic approach is illustrated in Figure 3, and the changes of Hb and Fib concentration are detailed in Figure 4. During two years of follow-up, he remained asymptomatic, without further bleeding episodes.

## Discussion

Diagnosing and managing OGIB is challenging due to its varied etiologies and often nonspecific symptoms. Nowadays, the development and application of new techniques, such as video-capsule endoscopy and device-assisted enteroscopy, have significantly improved the diagnosis rate of OGIB [3]. However, it remains a significant clinical challenge, especially in critically ill patients with acute OGIB who cannot tolerate prolonged or invasive endoscopic procedures and thus makes the treatment decision ambiguous. Digital subtraction angiography (DSA), while theoretically capable of detecting arterial bleeding rates ≥0.5 mL/min in the gastrointestinal tract [6], often yields negative results in acute cases [7]. Furthermore, delayed DSA performance and elevated platelet counts (>137 x 10^9^/L) have been associated with reduced sensitivity of DSA [8].

Current treatment for OGIB primarily involves endoscopic hemostasis or angiographic embolization [2]. For patients with severe bleeding where conventional diagnostic methods (including endoscopy, computed tomography angiography, and enteroscopy) fail to identify the bleeding source, or in cases where enteroscopy is not feasible, intraoperative endoscopy may be considered. However, this approach carries a high risk of complications and mortality, making it a last resort for refractory OGIB [9].

Long intestinal tubes are commonly employed for bowel decompression but have not previously been described as a route for localized hemostatic therapy. In our case, the patient presented with hemorrhagic shock and could not undergo enteroscopy. Despite comprehensive resuscitative efforts - including fluid replacement, transfusion, coagulation factor administration, and tranexamic acid - bleeding persisted. Thrombin, a well-established topical hemostatic agent, promotes clot formation by catalyzing the conversion of fibrinogen to fibrin. Yunnan Baiyao, a proprietary Chinese herbal formulation, has demonstrated procoagulant and anti-inflammatory properties in preclinical studies [10] and certain surgical settings [11]. The localized administration of thrombin and Yunnan Baiyao via a long intestinal tube resulted in rapid and sustained hemostasis in this patient, highlighting a novel therapeutic strategy. However, the accessibility of long intestinal tubes and Yunnan Baiyao may be limited in some healthcare settings, which could restrict the broader applicability of this approach.

This approach is not without risks. The potential for mucosal injury, bowel ischemia, or obstruction should be considered, and patients must be closely monitored. Furthermore, this report describes a single case with limited follow-up, and the generalizability of the approach remains uncertain. Further prospective studies are needed to evaluate its efficacy, safety, and broader clinical applicability.

## Conclusions

This case highlights the successful use of a long intestinal tube for localized hemostasis in acute, severe OGIB with massive hematochezia. Despite inconclusive diagnostic evaluations and persistent bleeding under aggressive medical management, targeted administration of thrombin and Yunnan Baiyao via the long intestinal tube achieved rapid and sustained hemostasis. While this approach may offer a promising alternative in cases where conventional interventions are not feasible, it remains an experimental strategy. Given that this case report describes a single case with limited follow-up, further prospective studies are necessary to evaluate its efficacy, safety, and clinical applicability.
